# Ethnic Differences in the Prevalence of Metabolic Syndrome: Results from a Multi-Ethnic Population-Based Survey in Malaysia

**DOI:** 10.1371/journal.pone.0046365

**Published:** 2012-09-28

**Authors:** Sanjay Rampal, Sanjiv Mahadeva, Eliseo Guallar, Awang Bulgiba, Rosmawati Mohamed, Ramlee Rahmat, Mohamad Taha Arif, Lekhraj Rampal

**Affiliations:** 1 Julius Centre University of Malaya, Department of Social and Preventive Medicine, Faculty of Medicine, University of Malaya, Kuala Lumpur, Malaysia; 2 Department of Epidemiology and Welch Center for Prevention, Epidemiology, and Clinical Research, Johns Hopkins University Bloomberg School of Public Health, Baltimore, Maryland, United States of America; 3 Department of Medicine, Faculty of Medicine, University of Malaya, Kuala Lumpur, Malaysia; 4 Department of Medicine, Johns Hopkins School of Medicine, Baltimore, Maryland, United States of America; 5 Area of Cardiovascular Epidemiology and Population Genetics, National Center for Cardiovascular Research (CNIC), Madrid, Spain; 6 Ministry of Health, Putrajaya, Malaysia; 7 Faculty of Medicine and Health Sciences, Universiti Sarawak Malaysia, Sarawak, Malaysia; 8 Faculty of Medicine and Health Sciences, Universiti Putra Malaysia, Selangor, Malaysia; The University of Queensland, Australia

## Abstract

**Introduction:**

The prevalence of metabolic syndrome is increasing disproportionately among the different ethnicities in Asia compared to the rest of the world. This study aims to determine the differences in the prevalence of metabolic syndrome across ethnicities in Malaysia, a multi-ethnic country.

**Methods:**

In 2004, we conducted a national cross-sectional population-based study using a stratified two-stage cluster sampling design (N = 17,211). Metabolic syndrome was defined according to the International Diabetes Federation/National Heart, Lung and Blood Institute/American Heart Association (IDF/NHLBI/AHA-2009) criteria. Multivariate models were used to study the independent association between ethnicity and the prevalence of the metabolic syndrome.

**Results:**

The overall mean age was 36.9 years, and 50.0% participants were female. The ethnic distribution was 57.0% Malay, 28.5% Chinese, 8.9% Indian and 5.0% Indigenous Sarawakians. The overall prevalence of the metabolic syndrome was 27.5%, with a prevalence of central obesity, raised triglycerides, low high density lipoprotein cholesterol, raised blood pressure and raised fasting glucose of 36.9%, 29.3%, 37.2%, 38.0% and 29.1%, respectively. Among those <40 years, the adjusted prevalence ratios for metabolic syndrome for ethnic Chinese, Indians, and Indigenous Sarawakians compared to ethnic Malay were 0.81 (95% CI 0.67 to 0.96), 1.42 (95% CI 1.19 to 1.69) and 1.37 (95% CI 1.08 to 1.73), respectively. Among those aged ≥40 years, the corresponding prevalence ratios were 0.86 (95% CI 0.79 to 0.92), 1.25 (95% CI 1.15 to 1.36), and 0.94 (95% CI 0.80, 1.11). The P-value for the interaction of ethnicity by age was 0.001.

**Conclusions:**

The overall prevalence of metabolic syndrome in Malaysia was high, with marked differences across ethnicities. Ethnic Chinese had the lowest prevalence of metabolic syndrome, while ethnic Indians had the highest. Indigenous Sarawakians showed a marked increase in metabolic syndrome at young ages.

## Introduction

Metabolic syndrome represents a constellation of risk factors, including raised blood pressure, dyslipidemia, central obesity and impaired fasting glucose, which reflect the underlying pathophysiology of insulin resistance [1]. Individuals with metabolic syndrome are at an increased risk of developing cardiovascular disease and diabetes [2]. With an estimated prevalence of 34% in the US and 10–50% in other countries depending on region, metabolic syndrome poses a major burden to modern society [3]–[5].

In Asia, the prevalence of metabolic syndrome has increased rapidly in recent years, partly as a result of rapid socioeconomic development [6], [7]. The prevalence of metabolic syndrome varies across Asian countries of varied economic prosperity, but it is uncertain if these differences are due to ethnic (with associated cultural practices), environmental or economic reasons [6], [8]–[10]. Malaysia is a rapidly developing country with a multi-ethnic heterogeneous population consisting of Malays (51%), Chinese (27%), Malay-related and aboriginal groups (11%), Indians (8%) and mixed ethnic groups (3%) [11]. Malays, Chinese and Indians form the majority of the population in West (Peninsular) Malaysia. In Sabah and Sarawak (East Malaysia), the two Malaysian States on the island of Borneo, most of the population is formed by indigenous groups such as Kadazans, Bajaus, Ibans, and Orang Ulu. Previous surveys of metabolic syndrome in Malaysia focused on specific groups or had a small sample size [12]–[16]. Our study is novel by providing detailed and generalizable comparisons of metabolic syndrome by ethnicity.

We conducted a large-scale population-based survey of all major ethnic groups in Malaysia to evaluate the prevalence of metabolic syndrome in the population, with specific emphasis in differences in prevalence across ethnic groups.

## Materials and Methods

### Study design

In 2004, we conducted a population-based cross-sectional survey of the Malaysian population aged 15 years and above. The Malaysian Statistics Department sampled the population using a stratified two-stage cluster sampling design. A detailed description of the sampling and data collection techniques has been previously reported [17]–[19]. A total of 18,805 subjects were interviewed giving an overall response rate of 93.2%. We excluded 1,594 subjects from the state of Sabah on the Island of Borneo (East Malaysia), representing 11.3% of the Malaysian population, due to study protocol violations as blood samples were not collected in the majority of households. This analysis thus included 17,211 individuals (7,306 men and 9,905 women). The Ethical Committees of the Ministry of Health Malaysia and the Faculty of Medicine and Health Science, Universiti Putra Malaysia reviewed and approved the study.

### Study Procedures

All study interviewers attended a central training 10-day workshop aimed to developing interpersonal communication skills; developing proficiency in using the study questionnaires and procedures; and instilling team building skills. All questionnaires, examinations and blood sampling procedures were performed at the participants' residences. Random checks for completeness of interviews were conducted on a daily basis in the field.

Information on self-reported ethnicity, socio demographic characteristics, personal and family history of disease, and use of anti-hypertensive and anti-diabetic was obtained using a pre-tested standardized questionnaire. A qualified nurse performed anthropometric and blood pressure measurements and collected fasting blood samples. Weight was measured to the nearest 0.1 kg using a digital scale (TANITA model HD 309). Height was measured by using a Body meter (SECA Model 208), which has a precision of up to 0.05 cm. Blood pressure was measured manually using a mercury sphygmomanometer. Two measurements were obtained in a sitting position and the average of two values were used in the analysis.

Fasting blood samples were collected using a standardized protocol after participants were instructed to fast for 12 hours prior to blood collection. Blood samples were kept in an icebox at all times, transported to the pathology laboratory (PATHLAB, Kuala Lumpur, Malaysia), and analyzed for serum glucose and lipids.

### Metabolic syndrome criteria

Metabolic syndrome was classified using the International Diabetes Federation/National Heart, Lung and Blood Institute/American Heart Association (IDF/NHLBI/AHA-2009) criteria as the presence of at least three of the following five risk factors: 1) Central obesity, defined for Asian populations as having a waist circumference ≥90 cm for males and ≥80 cm for females; 2) Raised serum triglycerides, defined as >1.7 mmol/L (150 mg/dL); 3) Low high density lipoprotein cholesterol (HDL-C), defined as <1.0 mmol/L (40 mg/dL) for males and <1.3 mmol/L (50 mg/dL) for females; 4) Raised blood pressure, defined as a systolic blood pressure ≥130 or a diastolic blood pressure ≥85 mmHg, or current use of anti-hypertensive medication; and 5) Raised fasting blood sugar, defined as ≥5.6 mmol/L (100 mg/dL) or current use of anti-diabetic medication [1].

### Statistical methods

Categorical variables were summarized as percentages with either standard error (SE) or 95% confidence interval (CI). Continuous variables were summarized as means with either SE or 95% CI. Robust Poisson regression was used to estimate marginally adjusted prevalences of metabolic syndrome for each ethnicity, as well as prevalence ratios for metabolic syndrome comparing each ethnicity to ethnic Malays as reference category. The associations between metabolic syndrome prevalence and ethnicity were adjusted using two multivariate models. Model 1 adjusted for age, gender and smoking status. Model 2 adjusted for the covariates in model 1 plus highest attained education, urban residence, family history of diabetes, and family history of hypertension. In addition, we performed detailed analyses of the shape of the dose-response relationship between age and metabolic syndrome and its components using restricted cubic splines with knots at the 10^th^, 50^th^, and 90^th^ percentiles of the age distribution.

Post stratification adjustments were performed for gender, ethnicity and age distribution differences between the sample and the total Malaysian population. We used multiple imputations with chained equations to address missing data in all analyses [20]. A total of 50 imputed sets were created, each a result of 200 iterations. Missing covariate patterns were individually explored for parsimony. Final imputation equations included sampling design covariates and all covariates from the final model. The residuals of the imputation regression models were graphically explored as a form of diagnostics. All analysis on imputed data accounted for the imputed data and for the complex survey nature of the datasets. A two-sided p-value<0.05 was considered as statistically significant. All data was analyzed using Stata 12.

## Results

The mean (SE) age of study participants was 36.9 (0.2) years with similar distribution of males and females (Table 1). A majority of the respondents were Malays (57.0%) followed by Chinese (28.5%), Indians (8.9%) and indigenous Sarawakians (5.0%). The mean waist circumference, serum triglycerides, HDL-C, and systolic and diastolic blood pressure of study participants were 81 cm, 1.5 mmol/L, 1.3 mmol/L, 122 mmHg and 79 mmHg. The distributions of age, smoking, urban residence, education, and family history of diabetes and hypertension, significantly differed across the ethnic groups (Table S1).

**Table 1:** Respondent characteristics by prevalence of metabolic syndrome.

Respondent characteristics	Overall	Metabolic Syndrome		P Values
		No	Yes	
	Mean ± SE or % (SE)			
Age	36.9±0.2	33.6±0.2	45.8±0.3	<0.001
Males	50.0 (0.4)	51.8 (0.5)	45.1 (0.8)	<0.001
Ethnicity				<0.001
Malay	57.0 (1.2)	57.8 (1.3)	54.8 (1.5)	
Chinese	28.5 (1.2)	29.0 (1.3)	27.1 (1.4)	
Indian	8.9 (0.5)	7.9 (0.5)	11.6 (0.8)	
Indigenous Sarawakian	5.0 (0.5)	4.8 (0.5)	5.5 (0.7)	
Others	0.7 (0.1)	0.5 (0.1)	1.0 (0.2)	
Current smoker	23.5 (0.4)	24.5 (0.5)	21.1 (0.7)	<0.001
Urban residence	70.1 (1.4)	69.8 (1.5)	71.0 (1.6)	0.264
Highest Education attained				<0.001
≥13 years (Tertiary)	14.4 (0.6)	16.3 (0.7)	9.3 (0.7)	
7–12 years (Secondary)	57.7 (0.6)	61.7 (0.7)	47.1 (1.0)	
≤6 years (No Formal and Primary)	28.0 (0.5)	22.0 (0.6)	43.6 (1.0)	
Family History of Diabetes	30.6 (0.5)	28.8 (0.6)	35.5 (0.9)	<0.001
Family History of Hypertension	47.5 (0.5)	45.8 (0.6)	52.0 (0.9)	<0.001
Physical measurement				
Body mass index, Kg/m^2^ (N = 14,578)	24±1	23±1	27±1	<0.001
Waist circumference, cm (N = 14,930)	81±1	77±1	91±1	<0.001
Systolic Blood Pressure, mmHg (N = 14,866)	122±1	118±1	134±1	<0.001
Diastolic Blood Pressure, mmHg (N = 14,866)	79±1	77±1	86±1	<0.001
Laboratory findings				
Glucose, fasting, mmol/L (N = 10,785)	5.6±0.1	5.1±0.1	6.8±0.1	<0.001
Total cholesterol, mmol/L (N = 11,144)	5.4±0.1	5.3±0.1	5.8±0.1	<0.001
Triglyceride, mmol/L (N = 11,143)	1.5±0.1	1.2±0.1	2.1±0.1	<0.001
HDL-cholesterol, mmol/L (N = 11,143)	1.3±0.1	1.3±0.1	1.1±0.1	<0.001
LDL-cholesterol, mmol/L (N = 11,093)	3.5±0.1	3.4±0.1	3.7±0.1	<0.001

Data on socio-demographic characteristics and co-morbidities were available on all 17,211 participants. Due to refusal, anthropometric characteristics, blood pressure and laboratory determinations were missing in some participants. Results in the table, however, reflect multiple imputation results for missing data incorporating complex survey characteristics (see Statistical Methods).

The overall prevalence of metabolic syndrome among all Malaysians aged 15 years and older was 27.5% (Table 2). The prevalence of metabolic syndrome was higher among Indians (35.6%) compared to Indigenous Sarawakians (30.5%), Malays (26.4%) and Chinese (26.2%). The prevalence of metabolic syndrome was higher among females (30.1%) compared to males (24.8%), and among participants ≥40 years of age compared to those 15 to <40 years.

**Table 2:** Prevalence of metabolic syndrome by gender among Malaysians ≥15 years, 2004.

Metabolic Syndrome	Prevalence, % (SE)		
	Females	Males	Total
Age ≥15 years			
All Ethnic Groups	30.1 (0.6)	24.8 (0.7)	27.5 (0.5)
Malays	29.0 (0.7)	23.7 (0.8)	26.4 (0.6)
Chinese	28.6 (1.2)	23.8 (1.4)	26.2 (0.9)
Indians	38.9 (1.7)	32.3 (2.0)	35.6 (1.5)
Indigenous Sarawakians	34.0 (3.1)	27.0 (3.0)	30.5 (2.4)
Age 15 to <40 years			
All Ethnic Groups	16.6 (0.6)	15.5 (0.8)	16.0 (0.5)
Malays	15.7 (0.8)	14.7 (0.9)	15.2 (0.6)
Chinese	13.9 (1.4)	13.4 (1.7)	13.7 (1.1)
Indians	23.3 (2.0)	21.8 (2.5)	22.5 (1.7)
Indigenous Sarawakians	26.3 (3.5)	22.1 (3.9)	24.2 (2.7)
Age ≥40 years			
All Ethnic Groups	50.5 (0.9)	38.7 (1.1)	44.6 (0.7)
Malays	51.5 (1.1)	38.5 (1.2)	45.0 (0.9)
Chinese	45.4 (1.7)	36.3 (1.9)	40.8 (1.3)
Indians	64.9 (2.4)	51.3 (3.2)	58.3 (2.2)
Indigenous Sarawakians	47.2 (4.5)	34.4 (4.6)	40.6 (3.4)

**Figure 1:**
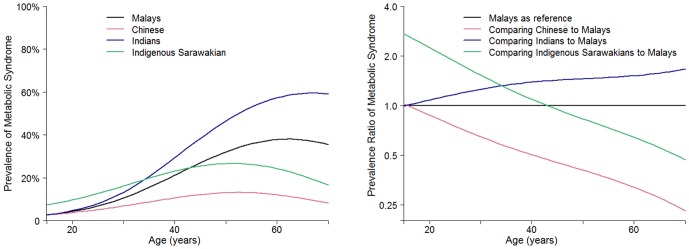
Age-variation in prevalence of metabolic syndrome by ethnicity. Prevalence estimates of metabolic syndrome and prevalence ratios were derived from restricted cubic splines models with knots at the 10^th^, 50^th^, and 90^th^ percentiles of the overall age distribution. Models were adjusted for age, gender, smoking status, highest attained education, urban residence, family history of diabetes, and family history of hypertension.

The crude and adjusted associations between the different ethnic groups and prevalence of metabolic syndrome are presented in Table 3. Compared to Malays, the prevalence of metabolic syndrome was 16% lower among Chinese (adjusted Prevalence Ratio, aPR 0.84; 95% CI 0.78 to 0.91), 31% higher among Indians (aPR 1.31; 95% CI 1.20 to 1.44) and 12% higher among the Indigenous Sarawakians (aPR 1.12; 95% CI 0.96 to 1.30). However, there was significant effect modification of the association between metabolic syndrome and ethnicity by age (Figure 1). Among those <40 years of age, the aPR for metabolic syndrome compared to Malays was 0.81 (95% CI 0.67 to 0.96) in Chinese, 1.42 (95% CI 1.19 to 1.69) in Indians, and 1.37 (95% CI 1.08, 1.73) in Indigenous Sarawakians. Among those ≥40 years of age, the aPR for metabolic syndrome compared to Malays was 0.86 (95% CI 0.79 to 0.92) in Chinese, 1.25 (95% CI 1.15 to 1.36) in Indians, and 0.94 (95% CI 0.80 to 1.11) in Indigenous Sarawakians. The P-value for the age by ethnicity interaction was 0.001.

**Table 3:** Association between metabolic syndrome and ethnicity by age.

	Crude Prevalence Ratio (95%CI)	Model 1*, Prevalence Ratio (95%CI)	Model 2*, Prevalence Ratio (95%CI)
Age ≥15 years			
Malay	1.00	1.00	1.00
Chinese	0.99 (0.91, 1.07)	0.87 (0.81, 0.94)	0.84 (0.78, 0.91)
Indian	1.35 (1.23, 1.48)	1.37 (1.26, 1.50)	1.31 (1.20, 1.44)
Indigenous Sarawakians	1.15 (0.98, 1.36)	1.11 (0.95, 1.29)	1.12 (0.96, 1.30)
Age <40 years			
Malay	1.00	1.00	1.00
Chinese	0.90 (0.75, 1.07)	0.83 (0.69, 0.98)	0.81 (0.67, 0.96)
Indian	1.48 (1.25, 1.76)	1.48 (1.25, 1.75)	1.42 (1.19, 1.69)
Indigenous Sarawakians	1.60 (1.26, 2.01)	1.40 (1.11, 1.76)	1.37 (1.08, 1.73)
Age ≥40 years			
Malay	1.00	1.00	1.00
Chinese	0.91 (0.85, 0.98)	0.89 (0.83, 0.96)	0.86 (0.79, 0.92)
Indian	1.30 (1.19, 1.41)	1.30 (1.20, 1.41)	1.25 (1.15, 1.36)
Indigenous Sarawakians	0.90 (0.76, 1.07)	0.93 (0.79, 1.09)	0.94 (0.80, 1.11)

* Model 1 adjusted for age, gender and smoking status. Model 2 adjusted for age, gender, smoking status, highest attained education, urban residence, family history of diabetes, and family history of hypertension.

**Figure 2:**
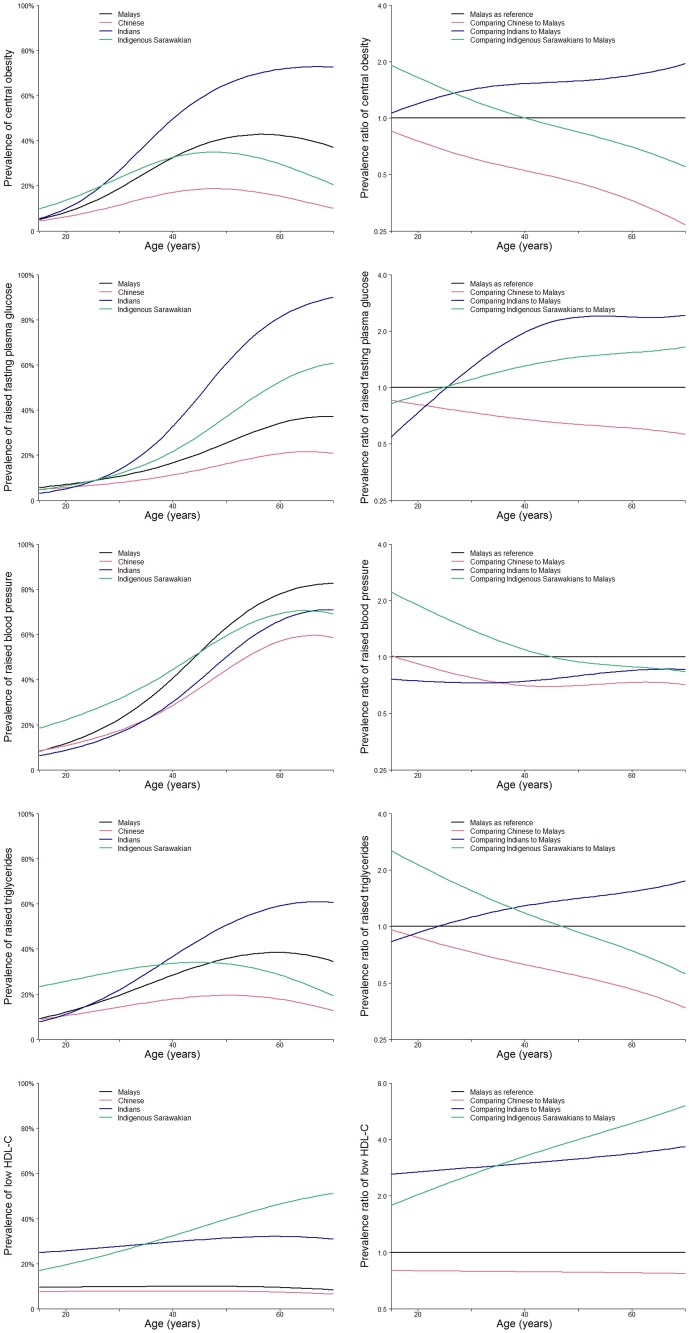
Age-variation in prevalence of various metabolic syndrome components by ethnicity. See Figure 1 for model details.

With respect to the individual components of the metabolic syndrome, the overall prevalence of central obesity, raised triglycerides, low HDL-C, raised blood pressure and raised fasting plasma glucose was 36.9%, 29.3%, 37.2%, 38.0% and 29.1%, respectively (Table 4). Figure 2 illustrates the association between ethnicity and the different criteria of metabolic syndrome. The association between ethnicity and central obesity, raised blood pressure and raised triglycerides across the different age groups followed the same pattern as for the association of age with metabolic syndrome: highest prevalence among Indigenous Sarawakians in younger age groups and among Indians in older age groups. The prevalence of raised fasting plasma glucose was similar in younger age groups among the different ethnic groups, but was higher in Indians compared to other groups in older age groups. Finally, the prevalence of low HDL-C was higher among Indians and Indigenous Sarawakians compared to the Malays and Chinese across all age groups.

**Table 4:** Prevalence of metabolic syndrome criteria by gender and ethnicity among Malaysians ≥15 years, 2004.

Metabolic Syndrome Criterion	Ethnic groups, % (SE)				
	Malay	Chinese	Indian	Indigenous Sarawakian	Total
Overall					
Central obesity	36.7 (0.6)	34.5 (1.0)	45.5 (1.4)	36.9 (2.2)	36.9 (0.5)
Raised triglycerides	29.2 (0.6)	27.9 (1.0)	32.0 (1.5)	33.7 (2.4)	29.3 (0.5)
Low HDL cholesterol*	35.7 (0.6)	32.8 (1.1)	52.1 (1.7)	53.2 (2.5)	37.2 (0.6)
Raised blood pressure	37.0 (0.6)	40.2 (1.1)	34.5 (1.2)	43.0 (2.7)	38.0 (0.5)
Raised fasting plasma glucose	28.3 (0.7)	29.7 (1.1)	38.3 (1.5)	17.1 (1.6)	29.1 (0.6)
Female					
Central obesity	46.3 (0.8)	41.3 (1.4)	55.7 (1.6)	47.4 (3.2)	45.8 (0.6)
Raised triglycerides	23.9 (0.7)	23.2 (1.2)	27.0 (1.6)	27.3 (2.6)	24.3 (0.6)
Low HDL cholesterol*	46.1 (0.9)	43.6 (1.6)	65.0 (1.9)	70.8 (2.6)	48.3 (0.7)
Raised blood pressure	35.1 (0.7)	36.1 (1.2)	30.6 (1.5)	36.6 (3.0)	35.0 (0.6)
Raised fasting plasma glucose	26.5 (0.8)	28.5 (1.2)	37.0 (1.7)	18.5 (2.2)	27.7 (0.7)
Male					
Central obesity	26.9 (0.8)	27.9 (1.3)	35.2 (2.0)	26.2 (2.6)	28.0 (0.6)
Raised triglycerides	34.5 (1.0)	32.5 (1.8)	37.1 (2.3)	40.1 (3.3)	34.4 (0.9)
Low HDL cholesterol	25.2 (0.9)	22.2 (1.6)	39.2 (2.3)	35.5 (3.1)	26.1 (0.8)
Raised blood pressure	38.9 (0.9)	44.2 (1.6)	38.4 (1.9)	49.5 (3.7)	41.0 (0.7)
Raised fasting plasma glucose	30.1 (0.9)	30.9 (1.6)	39.6 (2.4)	15.7 (2.4)	30.5 (0.8)

* HDL, indicates high density lipoprotein.

## Discussion

In this large population-based survey of the multi-ethnic Pan-Malaysian population, the overall prevalence of the metabolic syndrome was 27.5%, with higher prevalence in women (30.1%) compared to men (24.8%). Compared to ethnic Malays, the prevalence of metabolic syndrome was 16% lower among Chinese, 12% higher among Indigenous Sarawakians, and 31% higher among Indians. In addition, our data suggest that the association between ethnicity and metabolic syndrome was modified by age. Among younger participants, the distribution of metabolic syndrome was more tightly clustered among Malays, Chinese, and Indians, while Indigenous Sarawakians had a higher prevalence than the other ethnic groups. This difference among younger age groups may result, in the coming years, in a new ethnic distribution in the prevalence of metabolic syndrome in Malaysia.

The overall prevalence of metabolic syndrome observed in our study was lower than that reported in India and in Western Countries, similar to other reports from neighboring South East Asia countries, and is higher than reported rates in China. Using the same harmonized IDF definition of metabolic syndrome, the prevalence of metabolic syndrome was 31.4% in Kolkata, India [21], 23.3% in Thailand [22] and 7.3% in a large study from Guangdong, China [8]. Using the National Cholesterol Education Program, Adult Treatment Panel III (NCEP ATP III) diagnostic criteria, Bhardwaj et al identified a 45.3% prevalence of metabolic syndrome among 459 subjects in New Delhi, India [23], whilst a Chinese study of 15,540 subjects identified a 7.8% and 17.8% prevalence among men and women, respectively [6].

In our study, Indians had the highest overall prevalence of metabolic syndrome. It appears that Asians from the Indian sub-continent (i.e. South Asia) have a markedly higher prevalence of metabolic syndrome compared to the Chinese (i.e. East Asia). A higher prevalence of the metabolic syndrome among ethnic Indians residing in Western countries [24] and in smaller communities in Asia [13], [25] have been reported before. Ethnic Indians may be predisposed to several features of the metabolic syndrome. Compared to age-matched Caucasians of a similar body mass index and waist circumference, Indian adults, particularly females, have a higher percentage body fat, abdominal/central adiposity and subcutaneous adiposity [24], [26], [27], all of which are known determinants of insulin resistance. Furthermore, type 2 diabetes and non-alcoholic fatty liver disease (NAFLD), also related to the metabolic syndrome, occurs more frequently in Indians compared to other ethnic groups in Asia or in Western Countries [28], [29]. Lastly, cultural and lifestyle practices of ethnic Indians may further contribute to their higher prevalence of the metabolic syndrome. Compared to other ethnic groups, Indians have been shown to be less physically active on a daily basis [13], [24], and the Indian diet is traditionally high in carbohydrate and fat, with a lower fiber component, all of which may contribute towards insulin resistance and the metabolic syndrome [30].

The prevalence of metabolic syndrome among younger age groups in our study was higher among Indigenous Sarawakians compared to the other ethnic groups. For individual metabolic syndrome criteria, young Indigenous Sarawakians had higher prevalence of central obesity, raised blood pressure, raised triglycerides, and lower HDL-C compared to young participants of other ethnic groups. There is only limited information on the prevalence of cardiovascular risk factors in the state of Sarawak [16], [31], [32]. The Malaysia National Health Morbidity Survey III (NHMS III), conducted among subjects aged ≥18 years, reported that Indigenous Sarawakians had a higher prevalence of hypertension compared to other ethnic groups, but comparisons across ethnicities were not available for other metabolic syndrome components. Further research is needed to better explain the variation in the prevalence of metabolic syndrome among Indigenous Sarawakians as well as the evolution of metabolic syndromes at young ages.

Among older age groups, the prevalence of metabolic syndrome was higher in Malays compared to Chinese and Indigenous Sarawakians, but lower than in Indians. The prevalence of hypertension, among those aged >30 years in NHMS III was higher among Indians and Malays compared to the Chinese and Indigenous Sarawakian [32]. In the same survey, the prevalence of obesity was highest in Indians, followed by Malays and Chinese.

Apart from ethnic differences, our study also demonstrated age and gender differences in the prevalence of the metabolic syndrome, consistent with other Asian community-based studies [6], [8], [10], [22]. The higher prevalence of central obesity and low HDL-C, that explains some of the gender variation of metabolic syndrome, may be due to the higher prevalence of obesity and lower levels of physical activity among females. A higher prevalence of the metabolic syndrome among females compared to males is well recognized [33], partly due to an increase in the prevalence of the components of the metabolic syndrome at the peri-menopause and early menopause stage in females [34]. The cardiovascular risk profile of women can worsen at this time, possibly due to weight gain and changes in lipid profile. Regardless of gender, all components of the metabolic syndrome increase with age, likely due to time-related life style factors and diminishing physical activity with age [3].

Some limitations, of our cross sectional survey of the population of Malaysia in 2004, need to be considered. First, our cross-sectional design does not allow establishing causal statements. Second, it was difficult to attribute ethnic differences to more specific pathways. The association between ethnicity and disease observed in this study is a likely combination of genetic and environmental factors and their interactions along with temporal changes of society. Third, we excluded an ethnic sample from the state of Sabah (see Materials and Methods) and had a higher proportion of urban dwellers compared to the general population of Malaysia. However, there was appropriate representation of the major ethnic groups including Malays, Indigenous Sarawakians, Indians and Chinese, data on whom may be of relevance to other regions in Asia. The rigorous methodology, large sample size and representative educational levels of the study sample with respect to the general population are major strengths of our study.

We conclude that the prevalence of the metabolic syndrome in Malaysia, a multi-racial South East Asian population was 27.5%, a rate which is considerably higher than that of adults in East Asia but lower than that from South Asia (Indian sub-continent). Whilst rapid socio-economic development in Malaysia may be partly responsible for this pattern, we observed ethnic differences between ethnic groups, with ethnic Indians having a greater prevalence of metabolic syndrome compared to other ethnic groups. In addition, we found that the association between ethnicity and metabolic syndrome was modified by age. The distribution of metabolic syndrome among the different ethnic groups was more similar in the younger age groups compared to the older age groups, although young Indigenous Sarawakians had much higher prevalence of metabolic syndrome compared to other ethnic groups. It is thus critical to assess age-specific trends in metabolic syndrome among young and middle age groups in Malaysia, especially among indigenous populations. Further studies are also warranted to elucidate specific ethno-cultural related factors that may be intervened on in future prevention programs to reduce the burden of metabolic risk factors.

## Supporting Information

Table S1 Respondent characteristics by ethnicity.
(DOCX)
