# Supplementary material for: Ethnic Differences in the Prevalence of Metabolic Syndrome: Results from a Multi-Ethnic Population-Based Survey in Malaysia
Source: PLoS One. 2012 Sep 28;7(9):e46365. doi: 10.1371/journal.pone.0046365 (PMC3460855; doi:10.1371/journal.pone.0046365)
Supplement: Table S1 — Respondent characteristics by ethnicity. (DOCX) [file pone.0046365.s001.docx]

Table S1. Respondent characteristics by ethnicity

| Respondent characteristics | Malay | Chinese | Indian | Indigenous Sarawakian | P Values |
| --- | --- | --- | --- | --- | --- |
|  | Mean ± SE or % (SE) | | | |  |
| Age | 35.9 ± 0.2 | 39.6 ± 0.4 | 35.2 ± 0.4 | 37.0 ± 0.8 | <0.001 |
| Males | 49.7 (0.5) | 50.7 (0.8) | 49.9 (1.1) | 49.8 (1.9) | 0.73 |
| Current smoker | 27.2 (0.5) | 18.0 (0.8) | 15.6 (1.1) | 26.9 (2.0) | <0.001 |
| Urban residence | 60.8 (1.9) | 88.3 (1.8) | 85.9 (2.9) | 43.1 (7.0) | <0.001 |
| Highest Education attained |  |  |  |  | <0.001 |
| ≥13 years (Tertiary) | 13.4 (0.7) | 18.9 (1.2) | 10.9 (0.9) | 6.7 (4.0) |  |
| 7-12 years (Secondary) | 60.2 (0.7) | 52.0 (1.1) | 62.3 (1.2) | 51.9 (3.0) |  |
| ≤6 years (No Formal and Primary) | 26.4 (0.7) | 29.1 (1.1) | 26.8 (1.3) | 41.4 (3.3) |  |
| Family History of Diabetes | 30.6 (0.7) | 28.1 (1.0) | 49.6 (1.7) | 10.0 (1.9) | <0.001 |
| Family History of Hypertension | 50.1 (0.7) | 44.2 (1.1) | 45.0 (1.5) | 39.4 (2.8) | <0.001 |
| Physical measurement |  |  |  |  |  |
| Body mass index, Kg/m^2^ (N=14,578) | 24.2 ± 0.1 | 23.7 ± 0.1 | 24.3 ± 0.1 | 23.8 ± 0.3 | <0.001 |
| Waist circumference, cm (N=14,930) | 80.7 ± 0.2 | 80.9 ± 0.3 | 83.3 ± 0.4 | 80.0 ± 0.6 | <0.001 |
| Systolic Blood Pressure, mmHg (N=14,866) | 122.3 ± 0.2 | 122.9 ± 0.4 | 121.0 ± 0.5 | 124.8 ± 1.0 | <0.001 |
| Diastolic Blood Pressure, mmHg (N=14,866) | 79.0 ± 0.2 | 79.2 ± 0.2 | 78.5 ± 0.3 | 80.4 ± 0.6 | <0.013 |
| Laboratory findings |  |  |  |  |  |
| Glucose, fasting, mmol/L (N=10,785) | 5.6 ± 0.1 | 5.5 ± 0.1 | 6.1 ± 0.1 | 5.1 ± 0.1 | <0.001 |
| Total cholesterol, mmol/L (N=11,144) | 5.5 ± 0.1 | 5.4 ± 0.1 | 5.2 ± 0.1 | 5.3 ± 0.1 | <0.001 |
| Triglyceride, mmol/L (N=11,143) | 1.5 ± 0.1 | 1.4 ± 0.1 | 1.5 ± 0.1 | 1.6 ± 0.1 | 0.041 |
| HDL-cholesterol, mmol/L (N=11,143) | 1.3 ± 0.1 | 1.3 ± 0.1 | 1.2 ± 0.1 | 1.2 ± 0.1 | <0.001 |
| LDL-cholesterol, mmol/L (N=11,093) | 3.5 ± 0.1 | 3.4 ± 0.1 | 3.4 ± 0.1 | 3.4 ± 0.1 | <0.001 |

Data on socio-demographic characteristics and co-morbidities were available on all 17,211 participants. Due to refusal, anthropometric characteristics, blood pressure and laboratory determinations were missing in some participants. Results in the table, however, reflect multiple imputation results for missing data incorporating complex survey characteristics (see Statistical Methods).
